# Supplementary material for: Identification of a Novel Oxidative Stress- and Anoikis-Related Prognostic Signature and Its Immune Landscape Analysis in Non-Small Cell Lung Cancer
Source: Int J Mol Sci. 2023 Nov 10;24(22):16188. doi: 10.3390/ijms242216188 (PMC10671784; doi:10.3390/ijms242216188)
Supplement: Supplementary file 1 [file ijms-24-16188-s001.zip › ijms-2670665 Supplementary Resubmitted.pdf]

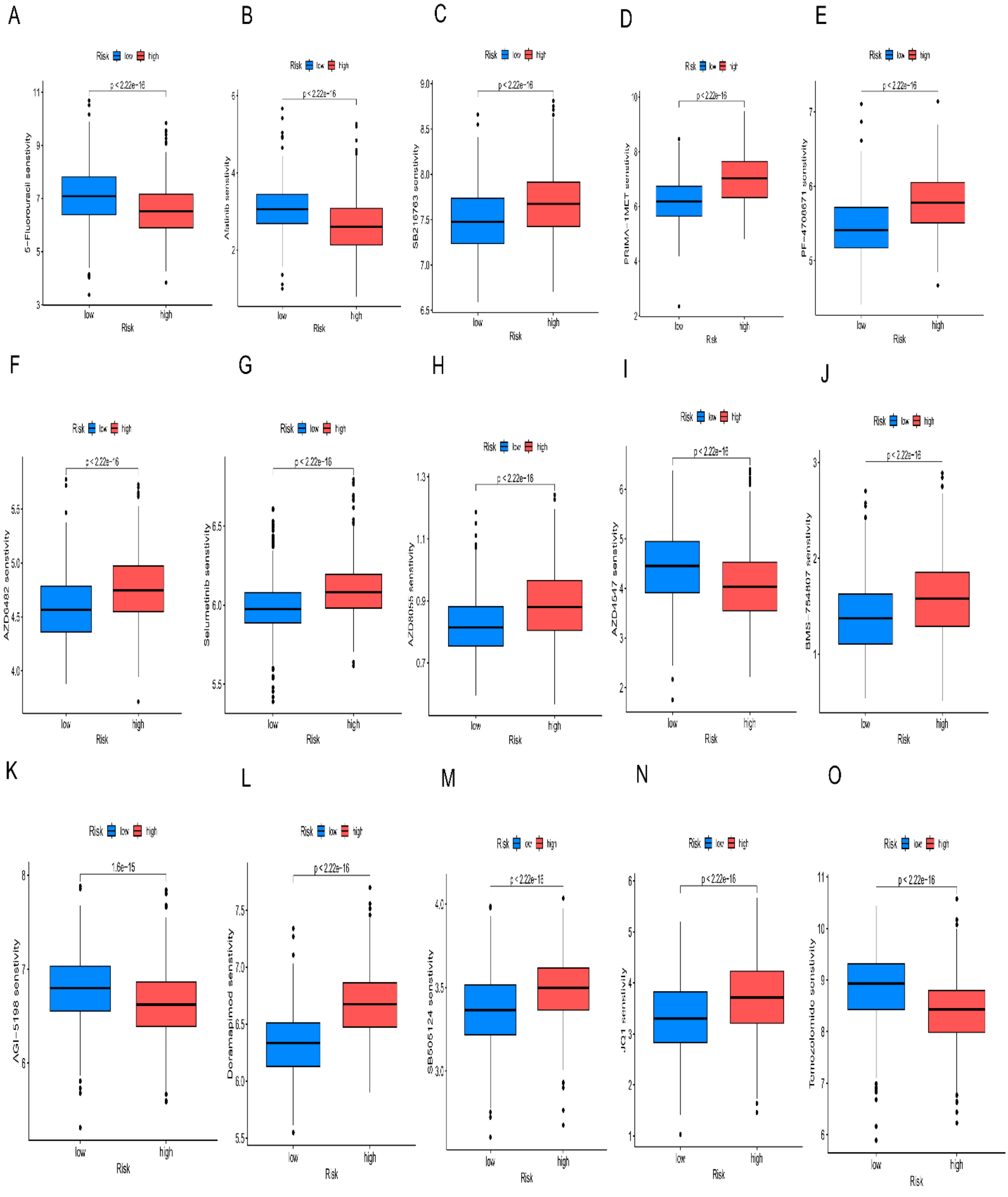


**Figure S1.** Drug sensitivity analysis

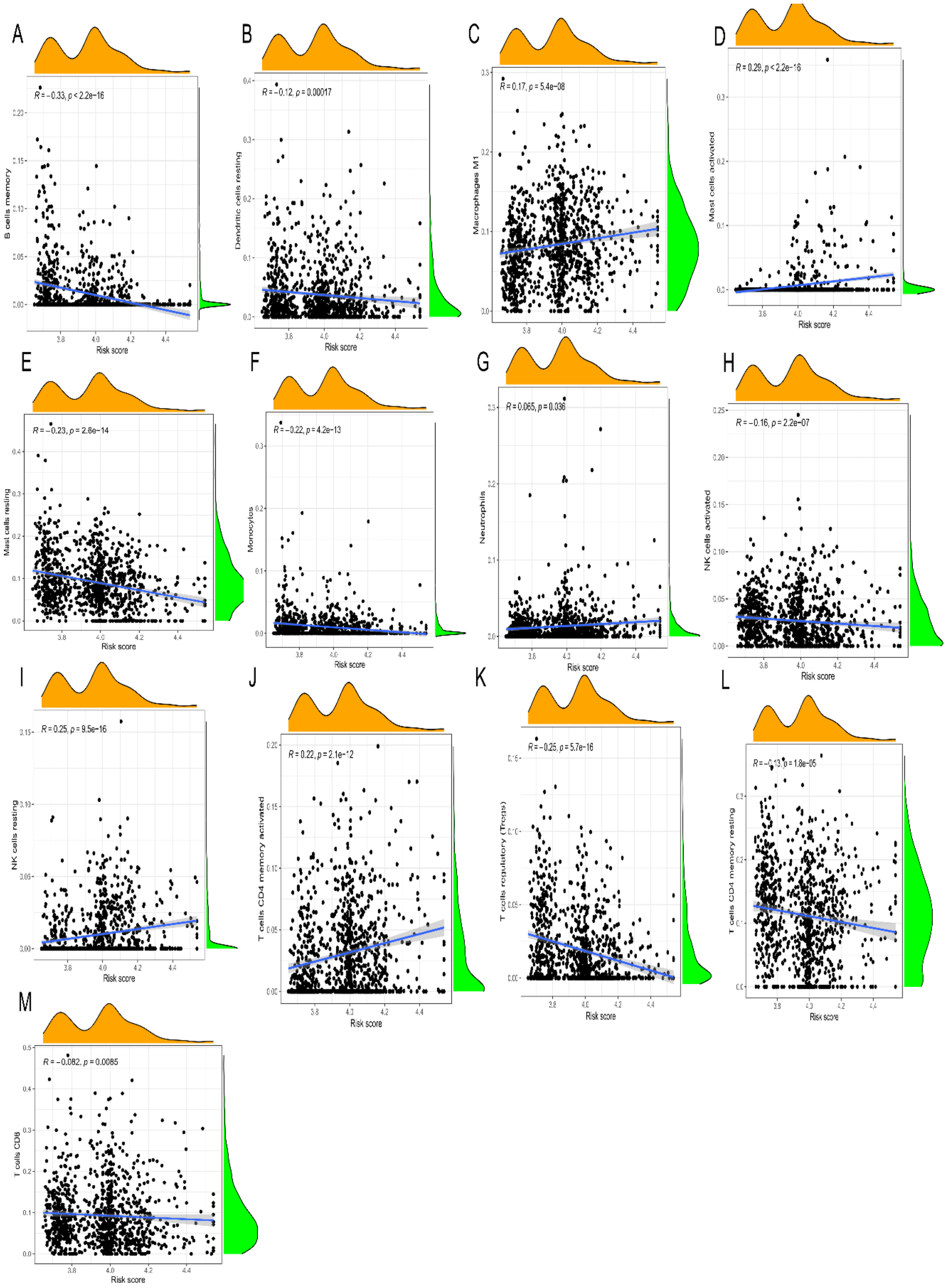


**Figure S2.** Association of immune cells other than m0 macrophages with risk scores

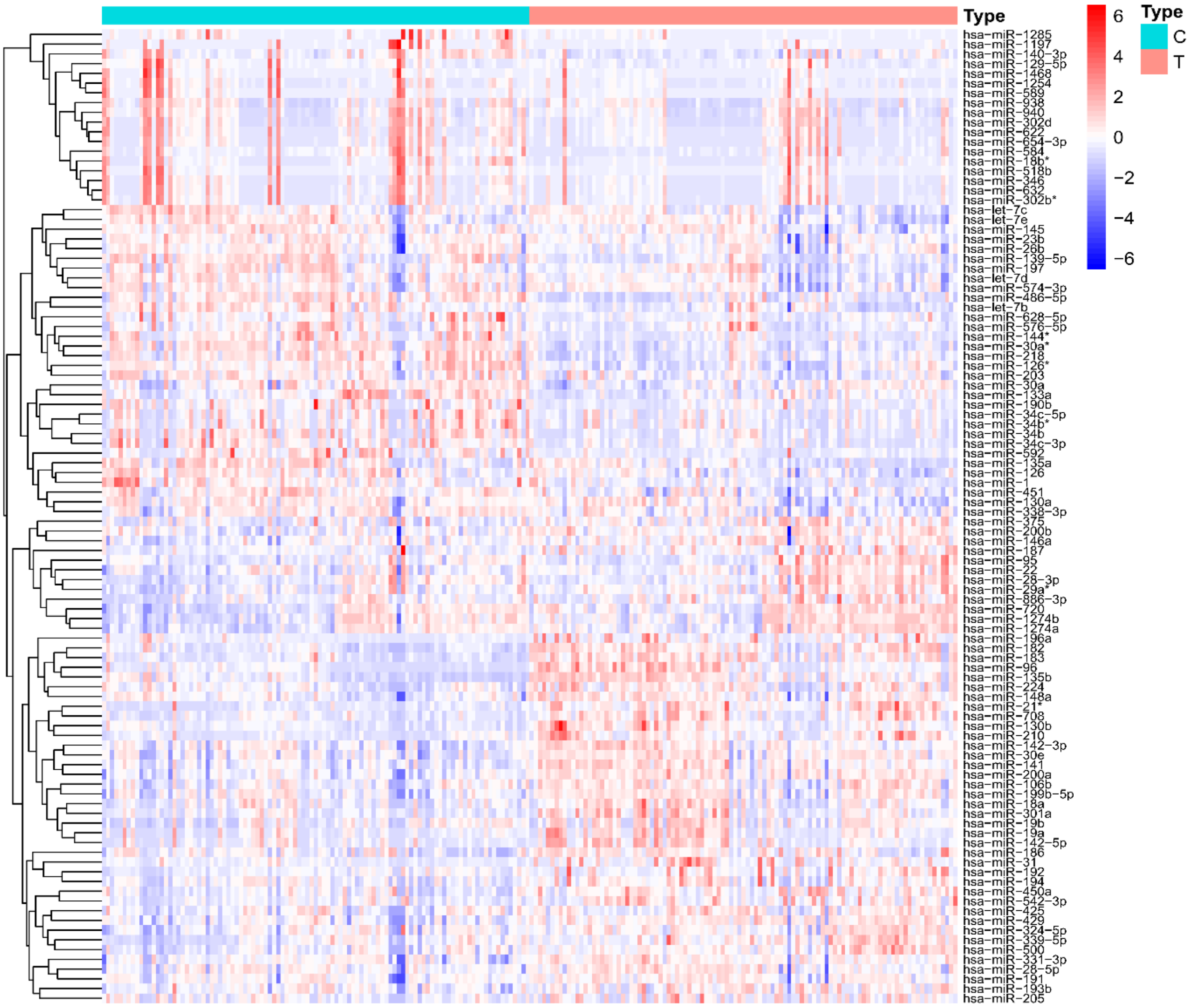


**Figure S3.** Differentially expressed miRNAs in NSCLC.

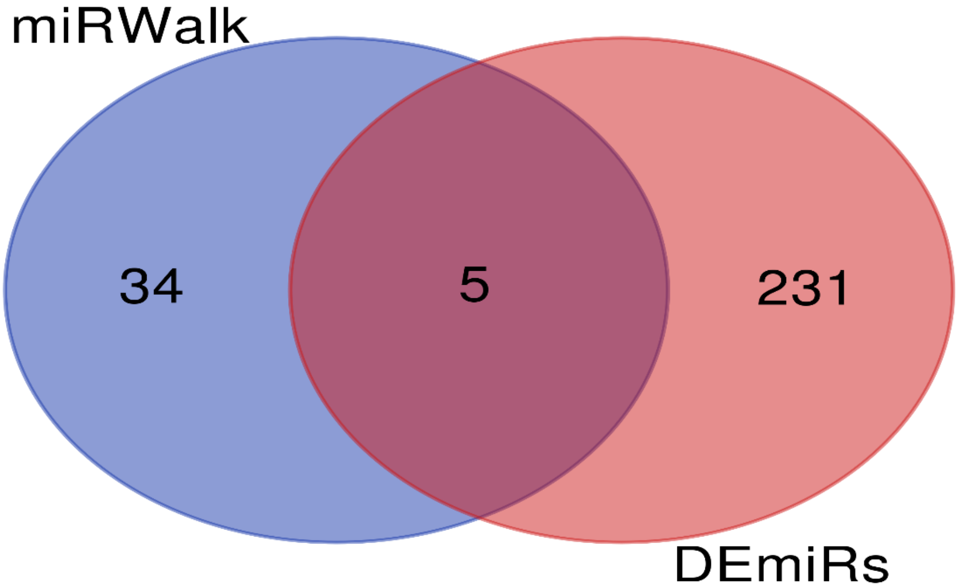


**Figure S4.** Venn diagram shows the intersection of miRNAs in the miRWalk database and differentially expressed miRNAs.
